# Enhancing Post-Operative Recovery in Spastic Diplegia through Physical Therapy Rehabilitation following Selective Dorsal Rhizotomy: A Case Report and Thorough Literature Analysis

**DOI:** 10.3390/children10050842

**Published:** 2023-05-06

**Authors:** Jawaria Shahid, Ayesha Kashif, Muhammad Kashif Shahid

**Affiliations:** 1 Department of Physical Therapy, Ikram Hospital, Gujrat 50700, Pakistan; itsjawaria@gmail.com; 2 Center of Physical Therapy, Rayan Medical Center, Gujrat 50700, Pakistan; 3 Department of Senior Health Care, Eulji University, Uijeongbu-si 11759, Republic of Korea; 4 Research Institute of Environment & Biosystem, Chungnam National University, Daejeon 34134, Republic of Korea; mkbutt2000@gmail.com

**Keywords:** selective dorsal rhizotomy, spastic diplegia, case report, spasticity, physical therapy, rehabilitation

## Abstract

Spasticity is a common issue among children, especially those with bilateral spastic cerebral palsy (CP). Selective dorsal rhizotomy (SDR) is a surgical procedure that is often used to decrease lower limb rigidity, alongside other treatment options such as intrathecal medication, peripheral nerve surgery, and deep brain stimulation (DBS). The objective of these therapies is to improve the standard of living for young individuals. This article intends to explain the motor deficits observed in spastic diplegia and a rehabilitation program using physical therapy after SDR. The information can help with counseling parents about the prognosis and developing a clinical treatment plan. The article presents a case study of a 12-year-old girl who recently underwent L3, L4, and L5 nerve root rhizotomy in the physical therapy department. It highlights the importance of long-term physical therapy follow-up and orthotic usage in the management of spastic diplegia.

## 1. Introduction

Spasticity refers to a motor dysfunction that involves elevated muscle tone and can severely affect the motor skills of young children [1]. It is considered to be the most prevalent type of motor impairment, particularly in conditions such as cerebral palsy (CP) [2,3]. Around 20 percent of individuals diagnosed with spastic diplegia, which is a type of cerebral palsy also known as bilateral spastic CP (BSCP) and classified as level V based on the Gross Motor Classification System (GMFCS), are identified as belonging to GMFCS levels I to IV. Studies have shown that individuals with BSCP experience a decline in their passive range of motion (PROM) in major lower limb joints between the ages of 2 and 14. BSCP also displays the most limited range of popliteal angle and knee extension when compared to other CP subtypes [4].

Spasticity is caused by imbalanced variables that work on raising and reducing muscle tone. It results from an abnormal increase and hypersensitivity of myotactic reflexes, and its severity is influenced by the speed of movement. While spasticity can be seen in multiple diseases which affect muscle tone and may arise spontaneously or as a result of brain dysfunction, athetosis, or dystonia, it is most commonly evident in CP [5].

In developed-country birth cohorts, the prevalence of CP is 1–2/1000 live births [6]. The incidence increases substantially with decreasing gestational age at delivery, reaching around a maximum of 100 per 1000 surviving children [7]. Spasticity in CP is often not evident right away. In fact, spasticity is frequently noticeable only after the first year of life in children with cerebral palsy [8]. Furthermore, spasticity often affects some muscle groups more than others [9]. For instance, Cacho et al. conducted an assessment of spasticity in various muscle groups and concluded that rigidity is more prevalent in the lower limb muscles when compared to the upper limb muscles [10]. Ghotbi et al. conducted a comparison study and found that ankle plantar flexor muscles are more susceptible to spasticity when compared to hip adductor muscles [11].

Several therapeutic options are being developed to treat spasticity, including deep brain stimulation, intraventricular baclofen (which enhances inhibitory activity on muscle tone), orthopedic procedures for dystonia, and rhizotomy [12,13]. Even after Botox injections, it is necessary to undergo physical therapy to enhance muscle strength and maximize range of motion (ROM) [14]. Botulinum toxin injections have been routinely utilized to treat spasticity in children with CP, with the ankle plantar flexor being the commonly targeted muscle [15,16,17].

The treatment of spasticity is essential in lowering muscular tone, enhancing quality of life, and reducing discomfort and deformities. If left untreated, spasticity can lead to chronic contractures, resulting in long-term shortening of muscle-tendon units that can affect functional skills such as standing, walking, and bed mobility, and can significantly impact the quality of life. Selective dorsal rhizotomy (SDR) is a proven surgical therapy for spastic diplegia. Although SDR has shown promising results for the treatment of spastic CP, its effectiveness across the entire spectrum of the condition and the long-term durability of its therapeutic benefits are still being studied. Spasticity is clinically classified into four types based on the affected limbs: spastic tetraplegia, spastic hemiplegia, spastic diplegia, and monoplegia [14].

The Ashworth Scale is a commonly employed assessment scale for spasticity [15,18]. Bryan Ashworth created the Ashworth Scale in 1964 as a tool for evaluating spasticity. The original Ashworth scale included five points, ranging from 0 to 4, with 0 representing no resistance and 4 representing a stiff limb in flexion or extension [15]. Subsequent modifications to the Ashworth Scale led to the development of the Modified Ashworth Scale (MAS) and the Modified Modified Ashworth Scale (MMAS). Ghothi et al. conducted a test–retest study to evaluate the variability and reliability of the MAS in measuring spasticity. The study found that the MAS is a highly reliable tool for assessing spasticity in the lower limbs [11]. According to Ansari et al., the MMAS proved to be a reliable tool for measuring knee extensor spasticity, as demonstrated by consistent measurements from a single rater and between raters [19].

**Figure 1:**
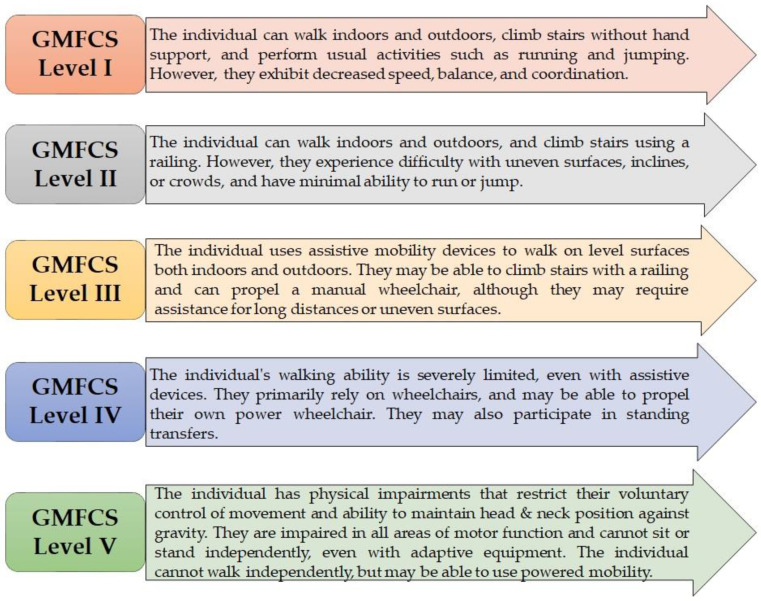
GMFCS levels in children.

Clinicians and academics have developed categorization methods based on a basic ordinal grading system of functional ability during the previous 20 years, allowing for more accurate communication between providers. The GMFCS is the firmly established and widely used functional categorization measure in CP [20]. The GMFCS is a five-level ordinal rating system designed to designate a person with CP’s gross motor function, as shown in Figure 1 [21]. Nordmark et al. indicated that, in children with CP, muscle shortening or contractures may have occurred at any point between the ages of 2 and 14 years [22]. The development of contractures could have varied depending on the child’s age and level of functional limitation, which could have been classified using the GMFCS within this age range. After conducting a long-term physical therapy follow-up of patients who underwent SDR, Annika et al. concluded that there was an improvement in gross motor function development post-operatively. The study found that patients in the age group of 2–14 years showed significant improvement (*p* value < 0.001) [4].

Motor impairments associated with spastic diplegia can have a significant impact on a child’s quality of life. To enhance lower limb function, SDR is a valuable surgical option, especially for young children who have bilateral spastic diplegia [5,23]. While SDR has shown promise in managing these impairments, there is limited research on the long-term effects of post-SDR physical therapy rehabilitation. In 1908, Foerster was the first to describe SDR, where he found that segmenting the dorsal (sensory) radicles can reduce spasticity and cause considerable muscular weakening as well as sensory and proprioceptive deficits [24]. Children with bilateral spastic CP undergo SDR, a neurosurgical technique, to lessen rigidity in their lower limbs. This procedure is primarily implemented at the lumbosacral level, and involves interrupting the monosynaptic stretch reflex afferent signal. To retain sensory and sphincter functions, the dorsal root is separated into radicles, and merely a fraction of these are sectioned [25]. 

This study focuses the literature on the long-term effects of post-SDR physical therapy rehabilitation for patients with spastic diplegia, highlighting specific interventions that have shown effectiveness in reducing spasticity and improving ROMs. A case study of a 12-year-old girl, who underwent SDR treatment and received physical therapy rehabilitation, is presented. The study emphasizes the significance of long-term physical therapy follow-up and the use of orthoses in treating spastic diplegia post-SDR, with the case study being the first reported in Gujrat, Pakistan. The findings of this study provide valuable insights into the long-term effects of physical therapy rehabilitation post-SDR and emphasize the need for effective management strategies for spastic diplegia in similar cases. The insights aim to provide a foundation for prognostic counseling with parents and planning clinical management for children with spastic diplegia post-SDR, underlining the importance of ongoing follow-up care. The novelty of this study lies in its emphasis on the significance of long-term follow-up and the use of orthoses in treating spastic diplegia post-SDR.

## 2. Materials and Methods

### 2.1. Literature Search

We conducted a comprehensive literature search to identify relevant publications published between 2000 and 26 April 2023, using systematic searches in various bibliographic databases such as PubMed, ScienceDirect, Scopus, and Web of Science. A total of 3756 papers were published in indexed journals in the Web of Science since 2001, consisting of research articles, case studies, reviews, and reports. Elsevier had the highest number of papers (975), followed by Taylor & Francis (494), Lippincott Williams & Wilkins (460), and Springer Nature (169). Other publishers such as Frontiers Media SA, Wiley, IOS Press, Foundation Rehabilitation Information, Sage, MDPI, Edizioni Minerva Medica, and Oxford University Press also contributed to the publications. To identify relevant recommendations, trials, systematic reviews, and meta-analyses, we used MeSH keywords such as ‘selective dorsal rhizotomy’, ‘spastic diplegia’, ‘rehabilitation’, ‘spasticity’, ‘physical therapy’, and ‘neurological injury’. Furthermore, we conducted a thorough review of the bibliographies of the identified studies to find pertinent articles while excluding duplicates.

### 2.2. Inclusion and Exclusion Criteria

To ensure a focused and relevant review of literature on improving post-operative recovery in spastic diplegia patients through physical therapy rehabilitation following selective dorsal rhizotomy, we established specific inclusion and exclusion criteria. Inclusion criteria comprised studies published between 2000 and 2023, in English, adhering to the PICO format, and reporting a diagnosis of spastic diplegia, impairments seen in daily living due to lower limb contractures, and those who underwent SDR. Additionally, studies on RCTs, systematic reviews, meta-analyses, and experimental studies were considered. Papers published in journals indexed in PubMed, ScienceDirect, Web of Science, and/or Scopus were also considered.

We excluded studies published before 2000, those that did not adhere to the PICO format, and publications such as book chapters, letters, proceeding papers, meeting abstracts, and book reviews. Non-English publications were also excluded. Studies with a risk of bias, those lacking sufficient data on effect sizes or statistical significance, and those not meeting our inclusion criteria were also excluded. Our exclusion criteria ensured that we focused on high-quality literature that meets our research questions and aimed to enhance post-operative recovery in spastic diplegia through physical therapy rehabilitation following selective dorsal rhizotomy.

### 2.3. Analysis of Co-Occurring Keywords

**Figure 2:**
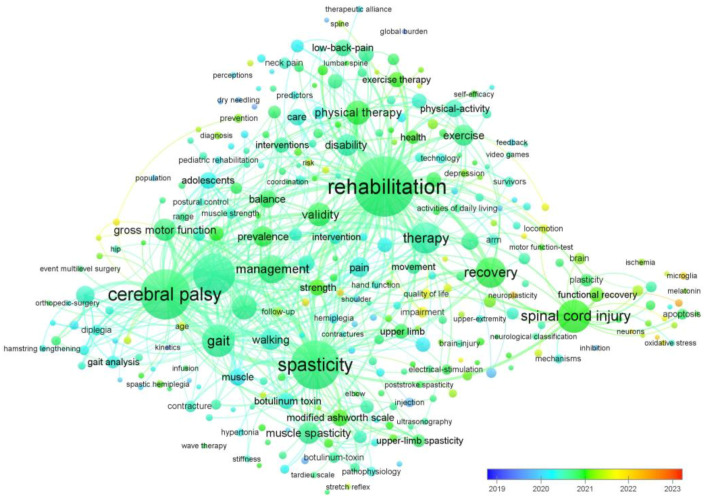
The co-occurring keywords in Web of Science indexed publications are displayed visually, with node size indicating the number of articles published and spot color representing the average publication time. Keywords displayed in blue represent earlier publications, while those displayed in red indicate relatively new keywords.

Using the open-source VOSviewer software, an analysis was conducted on 991 research articles (published between 2019–2023) to visualize the co-occurring keywords in Web of Science indexed journals. Figure 2 displays the results of the analysis, which heavily rely on visual elements. The analysis of only research papers published in Web of Science indexed journals from 2019–2023 shows that rehabilitation research was the most dominant field, with 54.19% of the 991 total research papers focusing on this area. Neurosciences, Sport Sciences, Orthopedics, and Pediatrics also received significant attention, with each field accounting for at least 15% of the total research papers (Table 1). It is possible that the actual number of unique research papers may be lower than the total count reported in this analysis due to potential duplication. However, without access to the individual papers and their specific categorizations, the exact extent of duplication in this dataset cannot be determined. Among the publishers, Elsevier was the highest contributor with 19.88% of the total research papers, followed by Taylor & Francis and Lippincott Williams & Wilkins. The results of this analysis suggest that there is a considerable interest in rehabilitation research, with a significant number of papers being published on this topic in the last five years. However, it is worth noting that the remaining percentage of papers may represent other important research areas that were not captured in this analysis.

### 2.4. Case Presentation

The 12-year-old girl was referred to the physical therapy department due to weakness and spasticity in her lower limbs. Her knees were bent inwards along with valgus deformity and her ankles were in equinus deformity, resulting in major balance impairments. She had a history of birth hypoxia and her mother had gestational insulin-dependent diabetes. Despite experiencing normal growth and development with no signs of delayed milestones, she gradually developed muscle stiffness that impaired her ability to walk independently. She was in GMFCS level V due to her inability to maintain anti-gravity posture and lower limb movements. After being diagnosed with spastic diplegia, various medical consultants attempted to treat the spasticity and prevent further muscle stiffness with medication, exercise, acupressure therapy, and injections. However, due to severe contractures and deformation, along with difficulty sitting and walking, she was referred to a neurosurgeon at the age of 12 who recommended SDR for muscle tone reduction. Following the procedure, she was referred to the physical rehabilitation facility 10 days after surgery for further care. We employed several evidence-based physical therapy interventions, including isometric contractions, stretching exercises, positioning strategies, standing with and without assistive devices, standing in parallel bars, walking in parallel bars, rocking board and wedge board, marching in a place, and cycling for strengthening.

## 3. Framework for Rehabilitation

The Physical Therapy Clinical Management Recommendations for Children with CP—Spastic Diplegia (PTCMR-SD) is a specialized program aimed at improving the functional mobility of children and adolescents with spastic diplegia [26]. The Guide to Physical Therapist Practice and International Classification of Functioning, Disability and Health (ICF) were utilized to develop a fundamental foundation for the PTCMR-SD and to recognize patient care components [27,28]. Creating a care plan is a complicated problem-solving activity that needs the integration of examination and evaluation findings as well as child and family goals. While designing a plan of treatment, the physical therapist should use elements of the ICF enablement model. Functional activities and engagement in life responsibilities should be the primary outcomes. For newborns, children, and adolescents with spasticity, there is a lack of evidence to help establish the ideal amount of intervention needed for the most successful functional results [29,30]. Bower and colleagues have demonstrated that a brief (2–3 weeks) intensive care results in short-term recovery in motor function at level III or below on the GMFCS age between 3 and 12 years, while, over a six-month period, there was no noteworthy difference between a higher (five times/week, 1 h session) and lower intensity of physical therapy [31]. Physical therapy after surgery should be rigorous, long-lasting, and always involve techniques to change the patient’s prior motor pattern [32]. The subject of service intensity requires more study. Table 2 provides an overview of several studies that have investigated different physical therapy protocols following SDR, including the types of exercises used. 

The rehabilitation process following surgery is essential, and, in certain cases, orthopedic correction procedures may also be required. In addition to improving bladder control and overall functionality, the inhibitory effect on ascending interneurons also has the potential to reduce stiffness in the upper limbs [33]. However, for newborns, children, and teenagers with cerebral palsy, there is insufficient evidence to establish the optimal level of intervention needed for optimal functional outcomes. Studies indicate that individuals who undergo SDR often require extensive physical therapy rehabilitation for around one year, starting in the days immediately after surgery and necessitating a hospital stay of six days to six weeks. Previous research has discussed the importance of pre-operative physical therapy [34]. Moreover, post-operative occupational therapy has also been highlighted in studies [35].

Studies have shown that patients who undergo SDR typically receive extensive physical therapy rehabilitation for about a year. The rehabilitation program starts on the first day after surgery, and patients usually spend 6–7 days in the hospital. Physiotherapy begins immediately after SDR, and includes early mobilization, range of motion exercises, and positioning strategies, such as sitting in different postures (e.g., supine lying, prone lying, side lying, and sitting with extended knees) [36,37]. Strengthening exercises for the quadriceps, hamstrings, abductors, and adductors, as well as practice for a normal gait pattern, are initiated within the first five days before discharge. Knee binders and KAFO are used to stimulate knee extension, and the exercises are carried out with functional or isolated control, progressive resistance training, and isolated training. The use of a parapodium is recommended after the first week [35,38].

**Table 2:** Characteristics of physical therapy protocols following SDR.

Author, Study Design	Post-Operative PT Start Day	Follow-Up Time (Year)	Physical Therapy Intensity and Frequency	Physical Therapy Interventions
Graubert et al. [39], prospective randomized trial	-	1	The patient underwent 4 weeks of therapy for 10 h per week, followed by 5 months of physiotherapy for 4–5 h per week, and then 6 months of PT for 1–3 h per week.	The patient underwent an intensive physical therapy program, which included the evaluation of gait kinematics and the improvement of ambulation.
Buckon et al. [35], RCT study	4th	1	The patient received two PT sessions daily and one OT session daily while in the hospital. After discharge, PT was conducted 3–4 times in a week and OT was conducted 1–2 times per week for a period of six months. PT continued for an additional year, with a frequency of 1–2 times per week.	The treatment plan included the use of assistive devices, occupational therapy, physical therapy, isometric contraction exercises, transfer training, and functional muscle strengthening.
Annika Lundkvist Josenby [4]	1st	10	For first 6 months post-surgery, the patients received one-hour sessions of therapy twice a week. Later on, the patients received therapy once a week for 18 months.	Primary goals of therapy were to improve the patient’s posture, enhance their balance control while sitting, standing, transferring, and walking, and also to train them on the use of assistive devices for walking.
Sophelia Hoi-shan Chan [38], case series study	2nd	1	At 6 and 12 months post-surgery, the patient underwent four weeks of therapy, consisting of 5 h per day.	The patient received both occupational therapy and physical therapy, with a focus on gait training.
Jack R. Engsberg [40]	-	2	From the 5th day to the 8th month post-surgery, the patient received therapy four times per week.	The therapy aimed to improve the patient’s gait speed and function, as well as cognitive skills.
Petra E. M. van Schie [37]	1st	1	During the first 3 months after surgery, the patient underwent therapy five times a week for 1 h each session. From the 3rd to the 6th month, therapy was conducted four times per week for one hour each session. From the 6th to the 12th month, therapy was conducted three times a week, with each session lasting 30 min.	The therapy program focused on improving the patient’s self-care abilities, gait training with specific emphasis on initial contact and heel-lift, and training in ADLs.
Annika Lundkvist Josenby [41], cohort study	1st	10	The patient received one-hour therapy sessions twice a week for 6 months, and then continued with once-a-week sessions thereafter for 18 months.	The therapy program included training the patient on functional ADLs, for example, getting in and out of bed, changing positions, and using assistive devices.

## 4. Results and Discussion

We have implemented an extensive exercise program with the aim of strengthening both the upper and lower extremities and improving the ROM of the muscles of the foot. Specific exercises that target the knee extensors, hip abductors, and hip extensors can be performed either individually or as part of natural movement patterns and postural control exercises. For instance, hamstring stretching, quadriceps strengthening, knee extension in prone and side-lying positions, hip flexion, and bridging exercises can lead to functional gains during 45 min daily sessions [42]. The physical limitations and stretching exercises used in the case study are described in Table 3. Once the patient’s base of support was deemed satisfactory, we typically began gait training in the second to third week of the exercise program. This training focused on typical motor patterns and utilized appropriate assistive devices to help improve the patient’s overall function and mobility [43].

**Figure 3:**
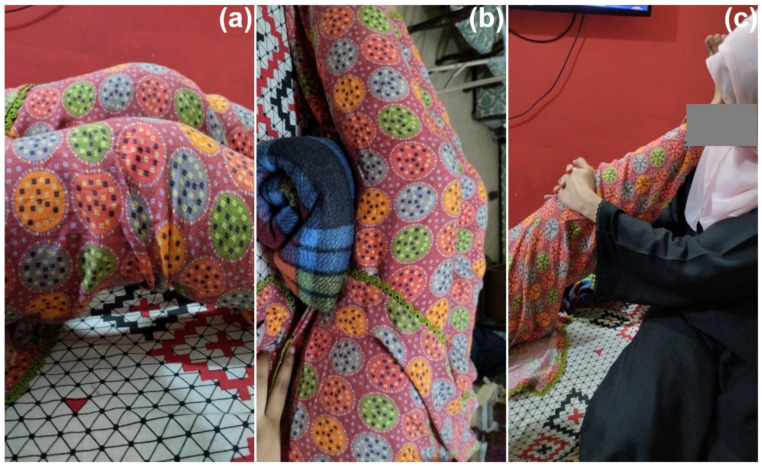
(**a**) Illustration of flexion contractures, (**b**) strengthening of hamstrings, and (**c**) assistance with knee joint extension.

Other strategies that were used to improve mobility and independence for patients with neurological disorders included training in postural transfer, with an emphasis on balance when sitting, kneeling, crawling, standing from a chair or the floor, and standing during gait exercises such as marching on the spot, half squats, wedge-board standing, parallel-bars standing, knee extension in standing, and walking exercises. Spider Therapy, as described in the program, enhances mobility and independence by using exercises in a spider cage to strengthen muscles, improve co-ordination, and enhance balance. The cage supports the patient in independent standing, allowing the therapist to easily instill proper posture and better body control [44]. The illustrations of flexion contractures, strengthening of hamstrings, and knee joint extension assistance are presented in Figure 3a–c, respectively.

**Figure 4:**
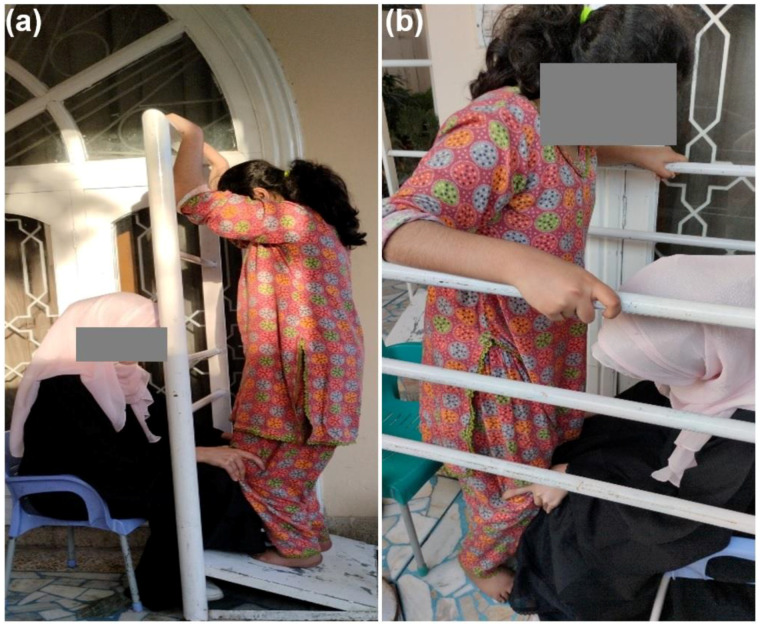
Illustrations of knee extension exercises with (**a**) wedge board and (**b**) parallel bars.

Figure 4a shows the patient standing on a wedge board while a physiotherapist stabilizes the knees and applies force to extend them. In Figure 4b, the patient is standing in parallel bars while a physiotherapist assists with extending the knees.

**Figure 5:**
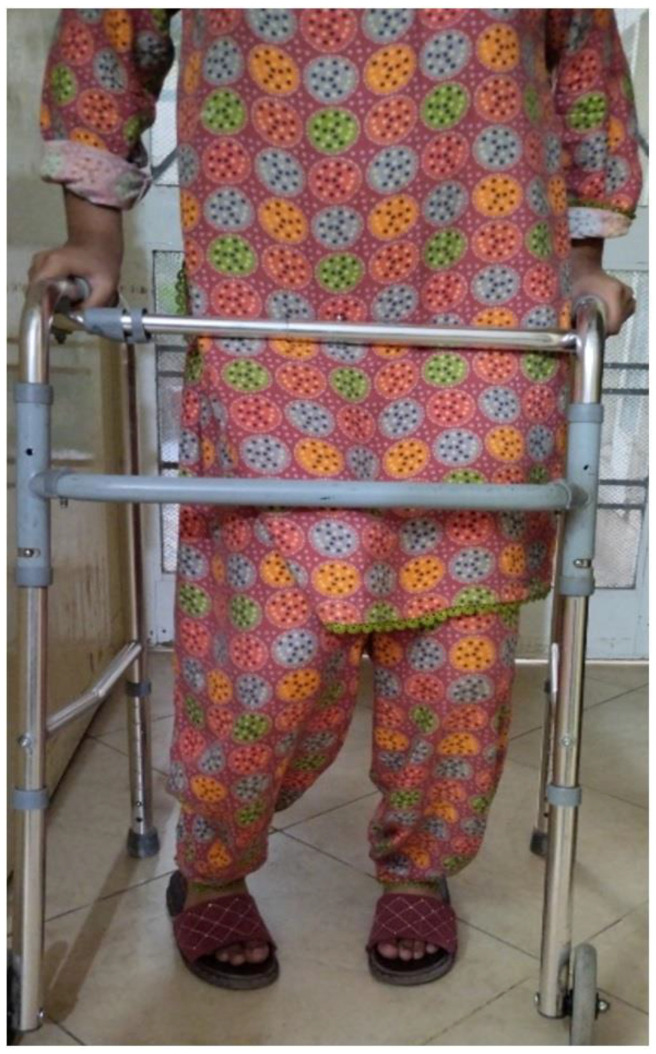
Illustration of indoor walking with hand-held mobility assistance.

Figure 5 demonstrates the patient walking indoors while using a hand-held mobility assistance device.

**Figure 6:**
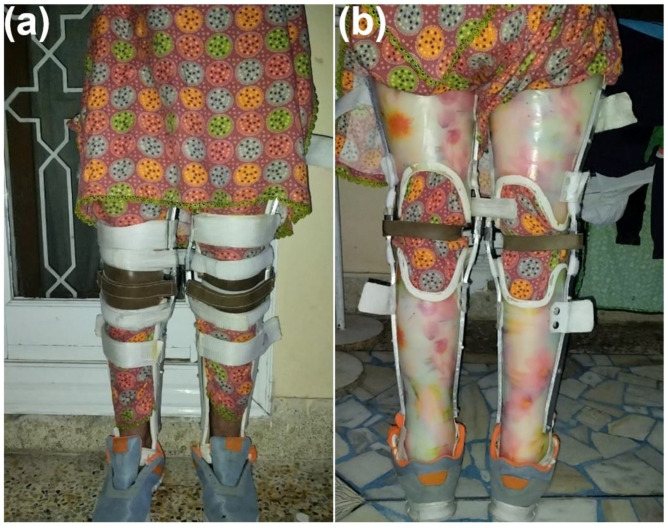
Knee–ankle–foot orthoses: (**a**) anterior and (**b**) posterior demonstration.

In order to reduce aberrant gaits and increase mobility for children with CP, lower extremity orthoses such as knee–ankle–foot orthoses (KAFOs) are utilized for ambulation, as shown in Figure 6a,b. With diplegia, KAFOs are not primarily employed for ambulation, but rather used to regulate aberrant movement during the stance phase of the gait and to assist weak muscles following procedures. The device is articulated at the sides of the ankle and extends from mid-thigh to the foot.

After undergoing SDR, the spasticity ratings in the lower limbs were significantly reduced. At two weeks post-surgery, the spasticity scores for the hamstrings were observed to be moderate to severe, while the right gastrocnemius muscles were mildly spastic (Table 4).

Table 5 presents the pre-operative ranges of lower limb motion and the ranges of motion for the lower limb during the physical examination conducted two weeks after discharge from the hospital and 8 months of physical therapy.

After 8 months of follow-up and long-term physical therapy, it was determined that the patient is now able to walk with assistance, but there is still 10 degrees of flexion while standing. The patient underwent physical therapy for one hour, five days a week for three months, and then for 45 min per day, five days a week. Following the long-term physical therapy, the patient was able to walk with a walker for one hour without experiencing fatigue.

We conducted a thorough analysis of the reported studies on the long-term benefits of physical therapy rehabilitation for patients with post-operative spastic diplegia, with a focus on specific interventions that have been shown to decrease spasticity and enhance ROM. Our findings demonstrate that combining SDR with long-term physical therapy and orthotic use leads to a remarkable improvement in muscle tone and motor skills. After an 8-month follow-up, we observed a significant change in GMFCS level from V to II. Furthermore, we found significant reductions in knee flexion contractures following the SDR and physical therapy protocol, which is consistent with previous studies [45]. We also observed significant decreases in ankle spasticity and improvements in ROM at the ankle, foot, and knee joints, which have also been reported in earlier studies [46].

Despite advancements in medical treatment, rehabilitation medicine, and surgical methods, spasticity in CP remains a significant barrier for individuals and professionals treating the condition [5]. Early surgical techniques, which were successful in lowering spasticity, were first reported by Sherrington in 1908 and subsequently developed and used by Foerster. In our study, we followed a patient undergoing a physical therapy program for one year after SDR. Our findings confirm that combining SDR with long-term physical therapy is a safe and effective method for treating spastic diplegia in children. When used in conjunction with physiotherapy, it improves gross motor function, functional skills and tasks, mobility, and self-care independence. These findings are consistent with current research [47,48,49].

The evidence-based interventions (as discussed in Section 2.4) have been found to yield greater results in reducing spasticity and improving quality of life [13,39]. The interventions were previously described in studies and had shown significant improvements. After 8 months of physical therapy, we observed a significant decrease in muscle tone, with the patient showing a flexion contracture of only 10 degrees compared to the initial 40 degrees on the first day of rehabilitation. Active and passive ankle dorsiflexion also increased by 6 degrees. Active straight leg raises (SLR) with knee extension is detected; abduction and adduction were improved by strengthening exercises. Wearing a KAFO for 16 h and an assistive stand for walking indoors and outdoors improved the gait pattern and reduced toe walking by the fifth month. Additionally, there was a decrease in the quantity of assistance required from walking aids. Significant reductions in lower extremity spasticity were also observed by Graubert et al. [39].

We encountered a few challenges during the study, such as difficulties in getting the patient to wear orthoses consistently and the patient gaining weight during rehabilitation. We recommend that clinical care guidelines be regularly updated to reflect the latest research and evolving approaches to treating and rehabilitating children with spastic diplegia. Further prospective trials with long-term rehabilitation follow-up procedures are advised. To clarify the indication criteria for SDR and determine the acceptability of current post-operative rehabilitation protocols, validated evaluation tools for investigating both static/functional characteristics and quality of life should be used. Further studies are needed to explore the impact of the SDR procedure on everyday functional activities and its long-term implications.

This case study can contribute to the understanding of how medical professionals can better treat individuals with bilateral spastic diplegia after SDR. Further studies on prospective trials with long-term rehabilitation follow-up procedures can more significantly define the outcomes and address any limitations of this study. Additionally, future studies in similar types of cases by other researchers can further fill the gap towards better understanding the efficacy of SDR combined with long-term physical therapy for reducing spasticity and improving gross motor function in children with spastic diplegia. Validated evaluation tools can also be used to investigate the impact of SDR on daily functional activities and quality of life. Considering the potential benefits of compensatory rehabilitation techniques, future studies may explore their effectiveness in improving functional outcomes and quality of life for individuals with neurological impairments [50,51]. In addition to other intervention strategies, deep breathing exercises have been incorporated to promote relaxation [52]. Such techniques may include adaptive strategies to compensate for specific deficits, training in the use of assistive technology, and cognitive-behavioral interventions aimed at promoting self-awareness and coping skills. Further research in this area can contribute to the development of more tailored and effective rehabilitation programs for individuals with neurological disorders. Overall, a continued effort to improve the treatment and rehabilitation of children with spastic diplegia is essential for achieving optimal outcomes and improving their quality of life.

## 5. Conclusions

In summary, this study highlights the crucial role of physical therapy in the rehabilitation of patients with spastic diplegia who have undergone SDR. It is essential to monitor patients’ neurological status during the procedure and provide ongoing rehabilitation with long-term follow-up. SDR has been shown to effectively reduce spasticity with minimal adverse effects and, combined with long-term physical therapy, can yield the most effective outcomes. Further research is needed to refine the rehabilitation protocols that follow the procedure and explore the potential of SDR as a treatment option for patients with spastic diplegia. Our study underscores the importance of a multidisciplinary approach to treating patients with spastic diplegia and highlights the critical role of physical therapy in their long-term care. 

## Figures and Tables

**Table 1:** Distribution of total research articles (991) published in 2019–2023 according to publishers and Web of Science categories.

Publisher	No. of Articles	Web of Science Categories	* No. of Articles
Elsevier	197	Rehabilitation	537
Taylor & Francis	146	Neurosciences	217
Lippincott Williams & Wilkins	86	Sport Sciences	200
Springer Nature	60	Orthopedics	188
Frontiers Media SA	48	Pediatrics	155
Wiley	47	Surgery	50
IOS Press	45		
Foundation Rehabilitation Information	43		
Sage	41		
MDPI	36		
Edizioni Minerva Medica	26		
Oxford Univ Press	23		
Others	193		

* Some research papers may fall into more than one Web of Science category, which can result in duplication or triplication of the number of articles.

**Table 3:** The physical limitations and stretching exercises employed in case study.

Physical Limitations	Suggested Stretching Exercises
Knee extension	Supine with therapist assisting extension, supine with heels pressing on a block and therapist apply force on knees to extend, fixed leg straps, supine with hip and knee extended, prone with weight hanging on ankle, wedge-board standing, standing in parallel bars
Ankle dorsiflexion	Standing, use of TheraBand, heels off step, calf stretch
Ankle plantarflexion	Frozen can roll, TheraBand stretch, balance-board-standing exercise
Inversion	Locomotor training while practicing loading and extension of lower limb, postural correction with both feet placed in anatomical position, standing balance exercise
Eversion	Side stepping, active stretches

**Table 4:** The spasticity ratings in the lower limbs. Categories for spasticity: (-) nil, (+1) mild, (+2) moderate, (+3) severe, and (>+3) more severe.

Muscle	Right	Left
Quadriceps	-	-
Hamstrings	+3	+3
Adductors	-	-
Gastrocnemius	+1	+1
TA	+1	+1
Extensor halluces longus	-	-

**Table 5:** Pre-operative and post-operative lower limb ranges of motion.

ROMs	Pre-Operative		Post-Operative (2 Weeks)		Post-Operative (8 Months)	
	Right (°)	Left (°)	Right (°)	Left (°)	Right (°)	Left (°)
Knee flexion	120	120	120	120	120	120
Knee extension limitation	90	80	40	40	10	10
Dorsiflexion	10	10	14	16	20	20
Plantarflexion	40	40	40	40	40	40
Inversion	30	20	20	20	20	20
Eversion	10	10	10	10	16	16

## Data Availability

All the relevant data are described in the main text.
